# Multilayer Black Phosphorus Near-Infrared Photodetectors

**DOI:** 10.3390/s18061668

**Published:** 2018-05-23

**Authors:** Chaojian Hou, Lijun Yang, Bo Li, Qihan Zhang, Yuefeng Li, Qiuyang Yue, Yang Wang, Zhan Yang, Lixin Dong

**Affiliations:** 1 A Key Laboratory of Microsystems and Microstructures Manufacturing, Ministry of Education, School of Mechatronics Engineering, Harbin Institute of Technology, Harbin 150001, China; houchaojian@163.com (C.H.); wyyh@hit.edu.cn (Y.W.); 2 Department of Electrical and Computer Engineering, Michigan State University, East Lansing, MI 48824, USA; libo2@msu.edu (B.L.); zhangq54@msu.edu (Q.Z.); yueqiuya@msu.edu (Q.Y.); 3 Department of Physics and Astronomy, Michigan State University, East Lansing, MI 48824, USA; lyfshishen1996@gmail.com; 4 School of Mechatronics Engineering, Soochow University, Soochow 215000, China

**Keywords:** black phosphorus, larger layer thickness, near-infrared photodetection, optoelectronic device

## Abstract

Black phosphorus (BP), owing to its distinguished properties, has become one of the most competitive candidates for photodetectors. However, there has been little attention paid on photo-response performance of multilayer BP nanoflakes with large layer thickness. In fact, multilayer BP nanoflakes with large layer thickness have greater potential from the fabrication viewpoint as well as due to the physical properties than single or few layer ones. In this report, the thickness-dependence of the intrinsic property of BP photodetectors in the dark was initially investigated. Then the photo-response performance (including responsivity, photo-gain, photo-switching time, noise equivalent power, and specific detectivity) of BP photodetectors with relative thicker thickness was explored under a near-infrared laser beam (*λ*_IR_ = 830 nm). Our experimental results reveal the impact of BP’s thickness on the current intensity of the channel and show degenerated p-type BP is beneficial for larger current intensity. More importantly, the photo-response of our thicker BP photodetectors exhibited a larger responsivity up to 2.42 A/W than the few-layer ones and a fast response photo-switching speed (response time is ~2.5 ms) comparable to thinner BP nanoflakes was obtained, indicating BP nanoflakes with larger layer thickness are also promising for application for ultra-fast and ultra-high near-infrared photodetectors.

## 1. Introduction

Recently, black phosphorus (BP) with puckered layers, became the focus of current research owing to its outstanding performance. Compared with other 2D materials, black phosphorus not only has higher mobility in the order of 10,000 cm^2^/(V·s) [1], but also has broadband photo-response owing to its thickness dependent direct bandgap ranging from 0.3 eV to 2 eV [2,3]. Therefore, BP-based photodetectors have great potential for future high-performance optoelectronic devices, especially in ultra-fast or ultra-sensitive photodetectors [4,5]. Until now, most efforts have been spent on improving the optoelectronic properties of BP photodetectors based on a monolayer or few layers. However, the major obstacle of monolayer or few layers BP for photodetectors is the environmental instability [6]. Compared with the monolayer or few layer BP nanoflakes, multilayer BP nanoflakes with thicker thickness have greater potential from both the fabrication viewpoint and the physical properties. For example, a protection strategy using a fully topmost oxidized BP layer as native capping in thicker BP nanoflakes was demonstrated for effectively secluding BP from water and preventing further degradation [7]. Furthermore, thicker BP nanoflakes have higher carrier mobility and a lower absorption cross-section than that of the monolayer BP nanoflakes [8]. Additionally, owing to its smaller bandgap, thicker BP nanoflakes have more obvious advantage in infrared photodetectors.

Based on above advantages, our report focused on the photo-response performance of multilayer BP nanoflakes with large layer thickness under near-infrared excitation. First, the thickness-dependence electron transport performance of BP Field Effect Transistors (FETs) was investigated. Then photo-response performance (including responsivity, photo-gain, photo-switching time, noise equivalent power, and specific detectivity) of BP photodetectors with relatively thicker thickness was explored by contrasting with previous work with few-layer ones. All the results show that the multilayer BP nanoflakes with large thickness have greater potential in future practical application.

## 2. Materials and Methods

### 2.1. Preparation of the BP Nanoflakes

**Figure 1:**
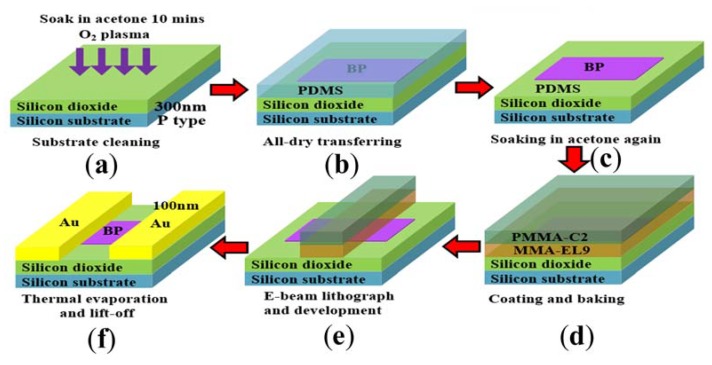
Schematic illustration of fabrication procedures of multilayer BP FETs. (**a**) Substrate cleaning by soaking in acetone and O_2_ plasma; (**b**) All-dry transferring; (**c**) Sample cleaning by soaking in acetone again; (**d**) Coating and baking e-beam lithography resist; (**e**) E-beam lithography and development; (**f**) Thermal evaporation and lift-off process.

In this typical experiment, the BP nanoflakes with different thicknesses (specifically 28 nm, 47 nm, and 302 nm) were obtained by repeatedly exfoliating bulk crystals using Scotch tape. An all-dry transfer technique [9] was used for transferring BP flakes onto a heavily doped p-type silicon substrate with 300-nm thick silicon dioxide. All substrates were cleaned in acetone for about 10 min, followed by O_2_ plasma cleaning before transferring (Figure 1a). In the all-dry transfer process (Figure 1b), polydimethylsiloxane (PDMS) was used as a viscoelastic stamp. To obtain the large-area BP nanoflakes, both BP nanosheets adhered with PDMS were baked on the hotplate for about 2 min at a temperature of 100 °C before exfoliating. Then the BP nanoflakes were mechanically transferred onto the PDMS. After that, under a Keyence Digital Optical Microscope and a micromanipulator, the BP nanoflakes were transferred onto the silicon substrate by peeling off the PDMS. Besides, prior to device fabrication, all samples were soaked in acetone for about 1 h to remove contamination (Figure 1c).

### 2.2. Fabrication of the BP FETs

Prepared samples were spin-coated with methyl methacrylate (MMA/EL9) and polymethyl methacrylate (PMMA C2) at 4000 rpm for 50 s (Figure 1d). Then the source/drain (S/D) electrodes were designed and patterned by e-beam lithography under Schottky field-emission scanning electron microscope (FESEM) from Hitachi SU5000, Tokyo, Japan. After development for about 40 s (Figure 1e) thermal evaporation technique was used for depositing Au electrodes (100 nm) under a vacuum of 2 × 10^−7^ Pa. Then the samples were soaked in acetone for about 2 h for the lift-off process (Figure 1f). After that, an annealing process was used to remove contaminations in vacuum at 400 K for 2 h.

### 2.3. Characterization and Measurements of BP FETs

**Figure 2:**
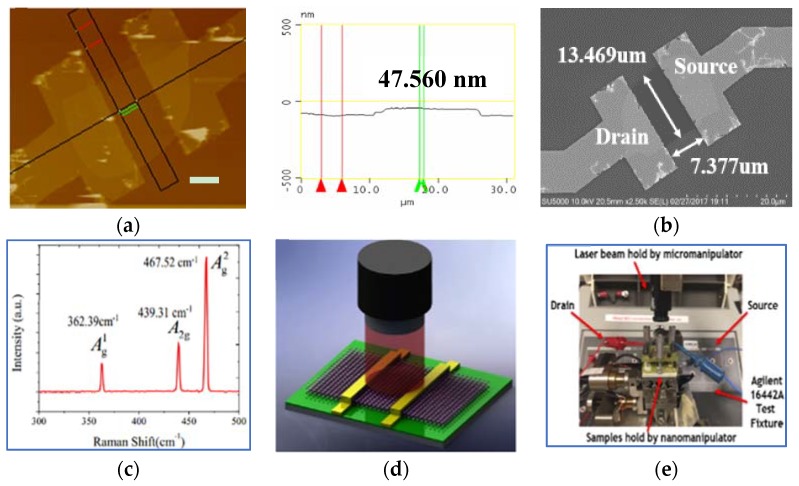
(**a**) The AFM image of one of the representative BP FETs. The scale bar is 5 μm. (**b**) The scanning electron microscopy (SEM) image of the same BP FETs. (**c**) Raman spectra of one of the representative multilayer BP nanoflakes used in (**a**). (**d**) Schematic of the multilayer BP phototransistors. (**e**) Physical picture of the multilayer BP phototransistors.

All of the BP nanosheets studied were cautiously selected under a microscope. As shown in Figure 2a, the thickness of one of the representative BP FETs is about 47.56 nm. The channel of the same BP FET has a 13 μm width and a 7 μm length (Figure 2b). Figure 2c shows the Raman spectrum of the same multilayer BP nanoflake. It can be clearly seen that there are three peaks representing pristine BP materials located at 362.39 cm^−1^, 439.31 cm^−1^, and 467.52 cm^−1^, respectively. The schematic and physical picture of the measurement setup of the BP photodetectors are illustrated in Figure 2d,e. An Agilent B1500A (National Test Equipment, Inc., Agilent, Santa Clara, CA, USA) semiconductor parameter analyzer was exploited for electrical characteristics. For characterization of optoelectronic performance, an infrared laser with a wavelength of 830 nm was irradiated vertically on the BP nanoflakes using a micromanipulator. The minimum spot size of the laser beam is larger than 120 μm for uniform irradiation. To obtain an accurate change of photocurrent, the sampling interval of the current signal was set at 4 ms.

## 3. Results

### 3.1. Thickness-Dependence Electron Transport Performance of BP FETs

**Figure 3:**
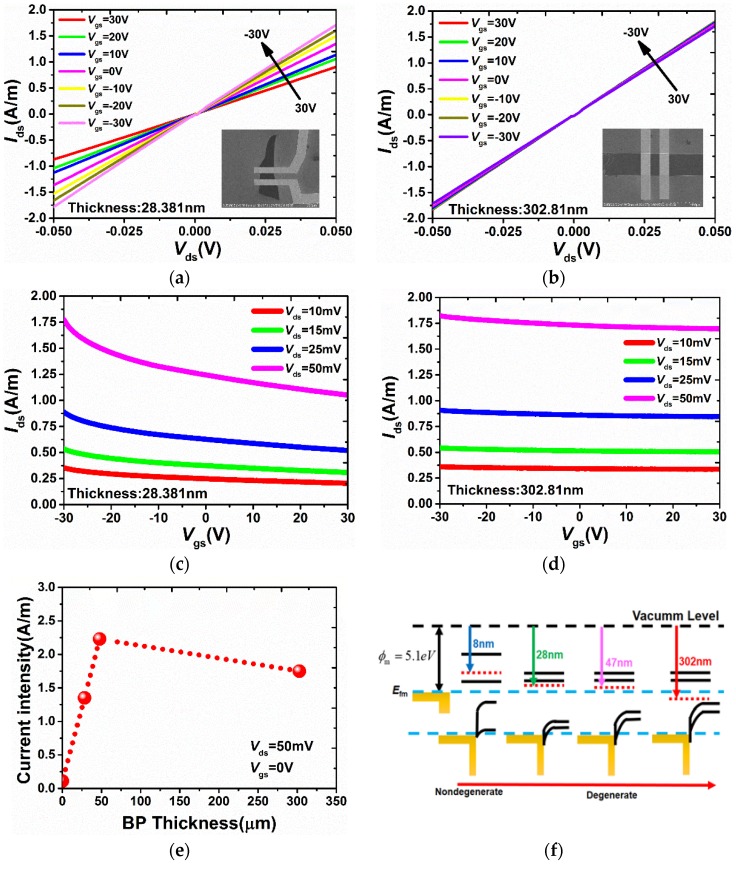
Output characteristics of BP FETs with (**a**) thickness of 28.381 nm and (**b**) with thickness of 302.81 nm. Inset: The SEM image of the same BP FETs. Transfer characteristics of BP FETs with (**c**) thickness of 28.381 nm and (**d**) thickness of 302.81 nm. (**e**) The current intensity as function of BP thickness. (**f**) Band diagrams for BP nanoflakes with different thicknesses.

The intrinsic output characteristics of as-fabricated BP FETs with small and large thickness (28.381 nm and 302.81 nm) in the dark are compared in Figure 3a,b. It can be seen that both devices show similar p-type feature, and the gate voltage-tunable output characteristics of the normalized current density (*I*_ds_/Width) in the thinner BP FETs is larger than that in the thicker one. At the same time, the comparison of transfer characteristics also shows that the current modulation of the gate voltage is weaker with the thickness increase, as shown in Figure 3c,d. In other words, the field-effect mobility (*μ*_eff_) decreases with the thickness increase. This phenomenon can be explained by a resistor network model that was used in multilayer graphene devices [10]. Due to the selected BP thickness being larger than the screening length of order ~10 nm [11], the finite interlayer conductivity *σ*_int_ is the main factor for lowering total mobility. More importantly, in order to exhibit a prominent ability to carry higher current intensity in the thicker BP nanoflakes, we compared our results with those of fewer-layer BP FETs with BP nanoflake thickness of 8 nm fabricated from Michele’s group through normalization of current intensity under the same applied voltage [5], all the data are presented in Figure 3e. The results indicated that the current intensity initially goes up with the thickness less than 47 nm, whereas current intensity slightly decreases in the much thicker BP nanoflakes.

To better understand this phenomenon, the detail investigations were as follows. From the transfer characteristics in Figure 3c,d, the BP nanoflakes selected in our study became degenerated p-type semiconductors with the Fermi level lying in the valence band, which is same as the results in [8]. In addition, the work function of BP tending to increase with flake thickness increase was demonstrated. Thus, band diagrams for BP nanoflakes with different thickness are shown in Figure 3f. All the positions of the Fermi levels were extracted from Liu’s group [12]. For thickness less than screening length (~10 nm), the device exhibits bipolar operation [5]. Even with the device in the on-state when *V*_g_ <*V*_FB_, the absence of screening from the substrate causes a large reduction of mobility, resulting in a lower current intensity (0.11 A/m). For our thicker BP nanoflakes, the depletion region decreases with the thickness increase into an optimal dimension (~47 nm) causing the larger current intensity (2.23 A/m), 18-fold larger than previous results. However, a slight reduction in terms of hole mobility for BP nanoflakes with thickness of 302 nm results in slight decrease of current intensity (1.75 A/m) for much thicker BP nanoflakes. In addition, this finding in terms of current intensity is commonly observed in the MoS_2_-based device with Pt electrodes [13]. Therefore, to eliminate the impact of screening on the device performance, it is more beneficial to exploit larger thickness in the device than single or few layer ones.

### 3.2. Photo-Response of Multilayer BP Photodetectors

To investigate the application of thicker BP nanoflakes for near-infrared photodetection, we compared the photodetection ability between the few-layer (under screening the length of BP nanoflakes is ~10 nm) and the thicker layer of BP nanoflakes. Based on this, there were two reasons considered in choosing the thickness of the BP nanoflake. First, a fully topmost oxidized BP layer as native capping in the thicker BP nanoflakes can effectively seclude BP from water and prevent further degradation. The selection of thicker BP nanoflakes is beneficial to prolong the lifetime of the device. Second, from the electron transport performance of BP FETs with different thicknesses, the electron transport property of devices between 47 nm and 302 nm is similar and the bandgap is almost 0.3 eV for BP nanoflakes with thickness above 28 nm. Therefore, the photo-response measurements were executed at a near-infrared excitation wavelength (830 nm) vertically irradiated on the BP nanoflake with thickness of 302.81 nm under *V*_ds_ = 200 mV. Additionally, it was demonstrated that the measured current increase in the BP devices by gate voltage cycles [5] and thus all the rest of the discussions about photo-response were carried out under zero gate voltage to exclude the gate-induced stress effect [14].

**Figure 4:**
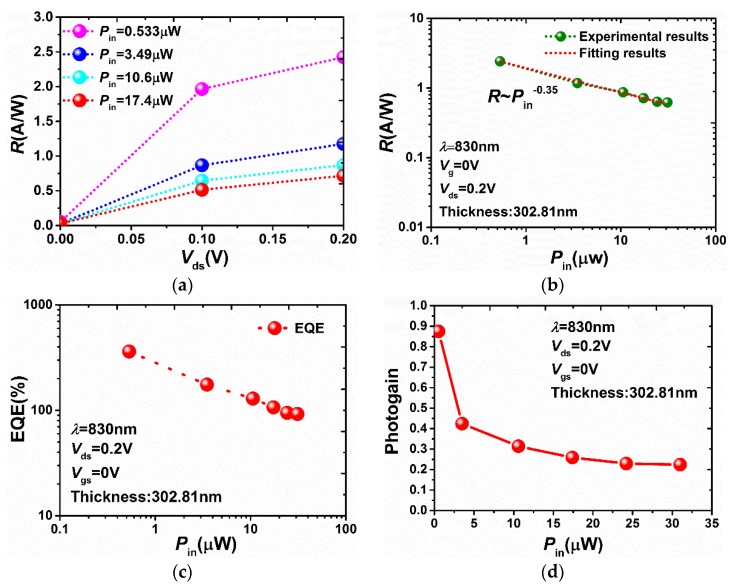
(**a**) *R* as a function of applied source-drain voltage under different optical powers. (**b**) *R* as a function of excitation power *P*_in_ incident on the device area. (**c**) *EQE* as a function of excitation power *P*_in_. (**d**) The photogain with a series of incident power.

**Figure 5:**
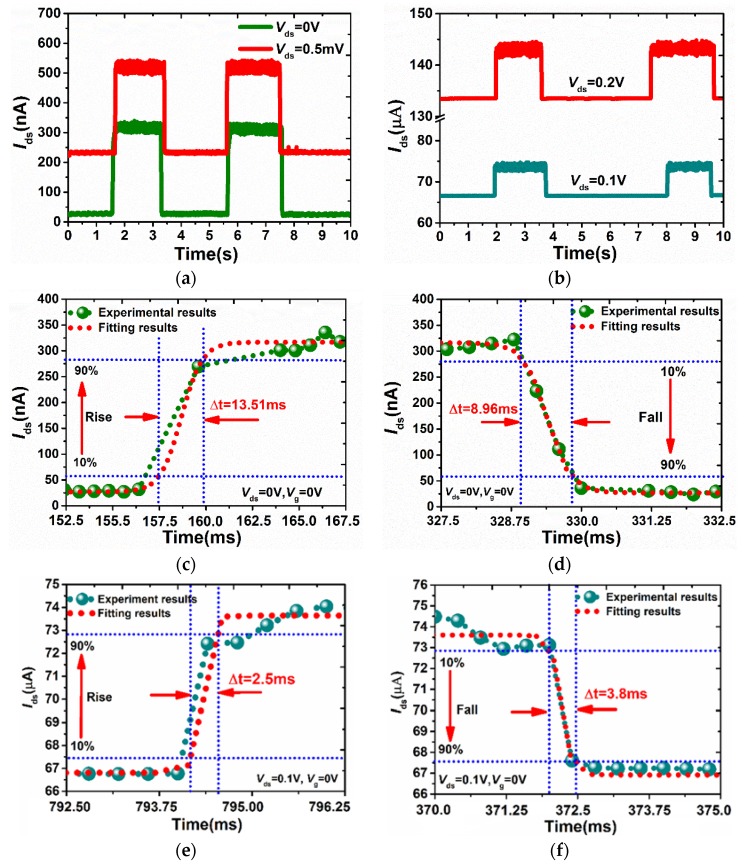
(**a**,**b**)The photo-switching performance under different bias voltages. (**c**)The rise process and (**d**) the fall process under *V*_ds_ = 0 V, *V*_g_ = 0 V, *P*_in_ = 10.6 μW. (**e**) The rise process and (**f**) The fall process under *V*_ds_ = 0.1 V, *V*_g_ = 0 V, *P*_in_ = 10.6 μW.

We first turned our attention to the responsivity (*R* = *I*_ph_/*P*_in_) of as fabricated BP FETs. Figure 4a shows *R* as a function of applied source-drain voltage under different optical powers. Note that the *R* gradually increase under the larger applied bias voltage, which is attributed to more effective separation of photocarriers under a larger electric field. Figure 4b shows *R* as a function of excitation power *P*_in_ incident on the device area. The responsivity decreases with the incident power increase. Moreover, by fitting the relationship between *R* and *P*_in_ with the power law, the exponent (~ −0.35) indicates that trap states and interactions among the photocarriers dominate the recombination. The maximum *R* is up to 2.42 A/W, which is far beyond the 8-nm BP photodetector 0.1 mA/W under the same conditions. The reason is mainly associated with reduction of bandgap that impacts on the energy of the photocarriers and the growth of the number of trap states that influences the lifetime of the photocarriers [15]. Additionally, external quantum efficiency (*EQE* = (*I*_ph_/*q*) × (*h*υ/*P*_in_) = *R* × (*h*υ/*q*)), as another important figure of the merit of photodetectors, was estimated to be approximately 360% under the illumination of laser with optical power around 0.533 μW. The *EQE* as function of optical power is shown in the inset of Figure 4c. A possible explanation of such a high *EQE* value is attributed to the multi-carrier generation results from the photon energy (~1.49 eV) being much larger than the energy bandgap (~0.3 eV). We further calculated the photo-gain (*G* = (*I*_ph_ ·*η*_ext_/e)/(*P*_in_/*h*υ)) given a value of *R* (Figure 5d). From the results, the maximum *G* operating under 830 nm is close to 0.9, which is larger than the near-infrared photodetector based on the MoS_2_/BP heterojunction [16].

To evaluate the photo-switching performance, the photo-switching performance under different bias voltages are shown in the Figure 5a,b. It can be seen that the current changed with the laser turned on or off. To accurately assess the speed of the device under laser irradiation, both the rise and fall processes were discussed in depth, as shown in Figure 5c–f. The rise time is defined as the time needed for a photodetector to go from 10% to 90% of the steady-state output, while the fall time is the opposite. The results show that our thicker BP photodetector exhibited a fast response, allowing one to respectively obtain the rise time and fall time to be 2.5 ms and 3.8 ms by fitting data of *V*_ds_ = 0.1 V, *V*_g_ = 0 V and *P*_in_ =10.6 μW. The fitting data was achieved by the Boltzmann function. However, the response time is slightly larger than that (1 ms) in the 8-nm BP photodetector [5]. This phenomenon is attributed to the origin of the photoresponse in thicker BP is dominated by the thermally driven thermoelectric and bolometric process [8]. Another apparent feature is that the response time under zero bias voltage is much larger than that with moderate bias voltage (0.1 V). The reason for this phenomenon can be deduced as follows. In fact, the total response time *τ* is related to three kinds of time, including intrinsic recombination time *τ*_r_ at very low bias voltage, the saturation time *τ*_s_ at larger bias voltage and the transit time *τ*_tr_ in the channel. The total response time can be written as [17]: *τ* = (*τ*_s_ + *τ*_tr_)^−1^ + *τ*_r_^−1^ and *τ*_tr_ = *l*^2^/2*μV*_ds_. Based on the above relationship, a moderate *V*_ds_ is beneficial to a reduction of the transit time *τ*_tr_ in the channel. Thus, both rise and fall time can be shown to have a great difference under different bias voltages. Additionally, the photo-switch performance for photodetectors based on other optoelectronic materials has a longer response time, such as the monolayer MoS_2_ (4000 ms) [18], In_2_Se_3_ (18 ms) [19], GaS (30 ms) [20], SnS_2_ (360 ms) [21], graphene [22] and nanowire photodetectors (580 ms) [23]. Therefore, it is easily demonstrated that the speed of photo-switch behavior of BP photodetectors is remarkable fast compared with other materials.

To further quantify the photo-response property of thicker BP photodetectors, both noise equivalent power (*NEP* = (2*eJ*_d_)^1/2^/*R*) and specific detectivity (D* = *RA*^1/2^/(2*eI*_d_)^1/2^) were studied to assess the sensitivity. Where *e* is electron charge, *J*_d_ is the current density in the dark, *R* is photoresponsivity, *A* is the effective absorption area. *I*_d_ is the current in the dark. The results were calculated from Figure 5c, leading to a *NEP* of 6.8 × 10^−11^ W/(cm Hz)^1/2^ and a D* of 1.833 × 10^8^ cm·Hz^1/2^/W. By comparison with *NEP* of previous MoS_2_ photodetectors (1.1 × 10^−10^ W/(cm·Hz)^1/2^) [24], our thicker BP photodetector is a more sensitive detector. However, the specific detectivity D* should be improved through comparison with other materials [25].

## 4. Conclusions

In conclusion, the intrinsic thickness-dependence electron transport performance of BP FETs in the dark was presented. The current intensity goes up with thickness less than a certain moderate thickness (~47 nm), whereas the current intensity slightly decreases in much thicker BP nanoflakes. This tendency is attributed to the screening effect in the thinner BP nanosheets and the interlayer conductivity in the thicker ones. Moreover, the responsivity up to 2.42 A/W show rise time and fall time to be 2.5 ms and 3.8 ms respectively indicating BP photodetectors with larger thickness are suitable fornear-infrared photodetection. Additionally, even though there is more sensitivity in terms of *NEP*, the lower specific detectivity of the thicker BP photodetector needs to be improved for an ultra-sensitive device. With these features, the multilayer BP FETs have great advantage in ultra-fast and ultra-high near-infrared photodetectors.

## References

[1] Yuichi A., Endo S., Narita S.I. (1983). Electrical properties of black phosphorus single crystals. J. Phys. Soc. Jpn..

[2] Qiao J., Kong X., Hu Z.X., Yang F., Ji W. (2014). High-mobility transport anisotropy and linear dichroism in few-layer black phosphorus. Nat. Commun..

[3] Kong L., Qin Z., Xie G., Guo Z., Zhang H., Yuan P., Qian L. (2016). Black phosphorus as broadband saturable absorber for pulsed lasers from 1 μm to 2.7 μm wavelength. Laser Phys. Lett..

[4] Huang M., Wang M., Chen C., Ma Z., Li X., Han J., Wu Y. (2016). Broadband Black-Phosphorus Photodetectors with High Responsivity. Adv. Mater..

[5] Michele B., Dirk J.G., Sofya I.B., Gary A.S., Ver der zant H.S.J., Andres C.G. (2014). Fast and broadband photo-response of few-layer black phosphorus field-effect transistors. Nano Lett..

[6] Island J.O., Steele G.A., Ver der zant H.S.J., Andres C.G. (2015). Environmental instability of few-layer black phosphorus. 2D Mater..

[7] Zhou Q.H., Chen Q., Tong Y., Wang J. (2016). Light-Induced Ambient Degradation of Few-Layer Black Phosphorus: Mechanism and Protection. Angew. Chem..

[8] Low T., Engel M., Steiner M., Avouris P. (2014). Origin of photoresponse in black phosphorus phototransistors. Phys. Rev. B.

[9] Gomez A.C., Buscema M., Molenaar R., Singh V., Janssen L., van der Zant H.S.J., Steele G.A. (2014). Deterministic Transfer of Two-dimensional Materials by All-Dry Viscoelastic Stamping. 2D Mater..

[10] Sui Y., Appenzella J. (2009). Screening and Interlayer Coupling in Multilayer Graphene Field-Effect Transistors. Nano Lett..

[11] Rodin A.S., Carvalho A., Neto A.H.C. (2014). Strain-Induced Gap Modification in Black Phosphorus. Phys. Rev. Lett..

[12] Liu X.C., Qu D.S., Li H.M., Moon I., Ahmed F., Kim C., Lee M., Choi Y., Cho J.H., Hone J.C. (2017). Modulation of Quantum Tunneling via a Vertical Two-Dimensional Black Phosphorus and Molybdenum Disulfide p−n Junction. ACS Nano.

[13] Das S., Chen H.Y., Penumatcha A.V., Appenzeller J. (2013). High performance multilayer MoS_2_ transistors with scandium contacts. Nano Lett..

[14] Wang H.M., Wu Y.H., Cong C.X., Shang J.Z., Yu T. (2010). Hysteresis of Electronic Transport in Graphene Transistors. Nano Lett..

[15] Bube R.H. (1992). Photoelectronic Properties of Semiconductors.

[16] Ye L., Li H., Chen Z., Xu J. (2016). Near-Infrared Photodetector Based on MoS_2_/Black Phosphorus Heterojunction. ACS Photonics.

[17] Youngblood N., Li M. (2017). Ultrafast photocurrent measurements of a black phosphorus photodetector. Appl. Phys. Lett..

[18] Oriol L.S., Lembke D., Kayci M., Radenovic A., Kis A. (2013). Ultrasensitive photodetectors based on monolayer MoS_2_. Nat. Nanotechnol..

[19] Gedrim R.B.J., Shanmugam M., Jain N., Christopher A.D., Michael T.M., Thomas M.M., Richard J.M., Richard L.M., Yu B. (2013). Extraordinary Photoresponse in Two-Dimensional In_2_Se_3_ Nanosheets. ACS Nano.

[20] Hu P., Wang L., Yoon M., Zhang J., Feng W., Wang X., Wen Z., Idrobo J.C., Miyamoto Y., Eeohegan D.B. (2013). Highly Responsive Ultrathin GaS Nanosheet Photodetectors on Rigid and Flexible Substrate. Nano Lett..

[21] Tao Y., Wu X., Wang W., Wang J. (2015). Flexible photodetector from ultraviolet to near infrared based on a SnS_2_ nanosheet microsphere film. J. Mater. Chem. C.

[22] Lai K.W.C., Xi N., Chen H., Fung C.K.M., Chen L. (2011). Development of graphene-based optical detectors for infrared sensing applications. IEEE Sens..

[23] Yoo J., Jeong S., Kim S., Je J.H. (2015). A stretchable nanowire UV–vis–NIR photodetector with high performance. Adv. Mater..

[24] Tsai D.S., Liu K.K., Lien D.H., Tsai M.L., Kang C.F., Lin C.A., Li L.J., He J.H. (2013). Few-Layer MoS_2_ with High Broadband Photogain and Fast Optical Switching for Use in Harsh Environments. ACS Nano.

[25] Wegrzecka I., Wegrzecki M., Grynglas M., Bar J., Uszynski A., Grodecki R., Grabiec P., Krzeminski S., Budzynski T. (2004). Design and Properties of Silicon Avalanche Photodiodes. Opto-Electron. Rev..

